# The virtual physiological human gets nerves! How to account for the action of the nervous system in multiphysics simulations of human organs

**DOI:** 10.1098/rsif.2020.1024

**Published:** 2021-04-14

**Authors:** A. Alexiadis, M. J. H. Simmons, K. Stamatopoulos, H. K. Batchelor, I. Moulitsas

**Affiliations:** ^1^ School of Chemical Engineering, University of Birmingham, Birmingham, Edgbaston B15 2TT, UK; ^2^ Strathclyde Institute of Pharmacy and Biomedical Sciences, University of Strathclyde, 161 Cathedral Street, Glasgow G4 0RE, UK; ^3^ Biopharmaceutics, Pharmaceutical Development, PDS, MST, RD Platform Technology and Science, GSK, David Jack Centre, Park Road, Ware, Hertfordshire SG12 0DP, UK; ^4^ Centre for Computational Engineering Sciences, Cranfield University, Bedford MK43 0AL, UK

**Keywords:** multiphysics, reinforcement learning, mathematical modelling of the intestine, virtual human, coupling multiphysics with artificial intelligence

## Abstract

This article shows how to couple multiphysics and artificial neural networks to design computer models of human organs that autonomously adapt their behaviour to environmental stimuli. The model simulates motility in the intestine and adjusts its contraction patterns to the physical properties of the luminal content. Multiphysics reproduces the solid mechanics of the intestinal membrane and the fluid mechanics of the luminal content; the artificial neural network replicates the activity of the enteric nervous system. Previous studies recommended training the network with reinforcement learning. Here, we show that reinforcement learning alone is not enough; the input–output structure of the network should also mimic the basic circuit of the enteric nervous system. Simulations are validated against *in vivo* measurements of high-amplitude propagating contractions in the human intestine. When the network has the same input–output structure of the nervous system, the model performs well even when faced with conditions outside its training range. The model is trained to optimize transport, but it also keeps stress in the membrane low, which is exactly what occurs in the real intestine. Moreover, the model responds to atypical variations of its functioning with ‘symptoms’ that reflect those arising in diseases. If the healthy intestine model is made artificially ill by adding digital inflammation, motility patterns are disrupted in a way consistent with inflammatory pathologies such as inflammatory bowel disease.

## 1. Introduction

In Mary Shelley's novel, Dr Frankenstein brings his creature to life by, in line with the contemporary theory of galvanism, pumping electricity into the creature's nervous system. In fact, the ability of the nervous system to receive and respond to external stimuli has always been recognized as an essential manifestation of life. Away from galvanism, today scientists pursue the objective of bringing (digital) the so-called *virtual physiological human* to life [1], a computer analogue of the human body where new treatments, bold medical hypotheses and even disrupting ideas can be tested in a safe environment. This is the ultimate goal of *in silico* medicine: integrating computer models of the mechanical, physical and biochemical functions of the living human body into the virtual physiological human.

One of the obstacles to achieving this goal remains our difficulty to replicate the activity of the autonomic nervous system (ANS) within multiphysics models. Human physiology is not the mere result of the well-known laws of physics and chemistry but responds dynamically to environmental stimuli to ensure the correct functioning of the body. This ability, known as homeostasis, is regulated by the ANS that adjusts the response of the organism to the perception of the environment [2].

Computational neurosciences provide several models of neurons and neural systems [3]. However, there is still a long way before these models can be integrated into multiphysics simulations. From a certain point of view, the state of our virtual human remains vegetative. *In silico* hearts only beat with fixed rhythms [4,5], *in silico* lungs only breath with immutable frequencies [6,7], and *in silico* intestines only contract with predetermined patterns [8,9].

Alexiadis [10] showed that artificial intelligence (AI) can enhance multiphysics simulations. In Alexiadis *et al.* [11], a multiphysics model of the intestine coupled with an artificial neural network (ANN) could learn autonomously how to coordinate its contractions and propagate the luminal content in a given direction (peristalsis). The model could *learn* peristalsis, but it could not *adapt* peristalsis to the physical properties of the luminal content. The ANN used in that study was ‘omniscient’. It had perfect knowledge of the environment, which does not correspond to the actual capacity of the enteric nervous system (ENS) to sense the luminal environment. In this study, we show that the ANN should not be omniscient, but it should carefully replicate the input–output structure of the ENS. Moreover, in multiphysics simulations, diseased states are usually hardcoded into the model [5,12–15]. This study introduces a multiphysics + ANN model of the healthy intestine that, under certain conditions, ‘becomes diseased’ without the need to hardcode the disease state into the model. In fact, if (digital) inflammation is added to the intestinal walls, the motility pattern of the healthy model is disrupted in a way consistent with inflammatory pathologies such as inflammatory bowel disease (IBD).

## 2. Results and discussion

### 2.1. The multiphysics model without an artificial neural network (non-adaptive model)

The multiphysics model is based on discrete multiphysics (DMP) [16] and combines two-particle methods: smooth particle hydrodynamics (SPH) to model the fluid [17] and the lattice spring model (LSM) to model the elastic membrane [18]. This is the baseline against which the adaptive model will be compared; the Methods section provides details on how SPH and LSM are implemented in the model.

**Figure 1.**
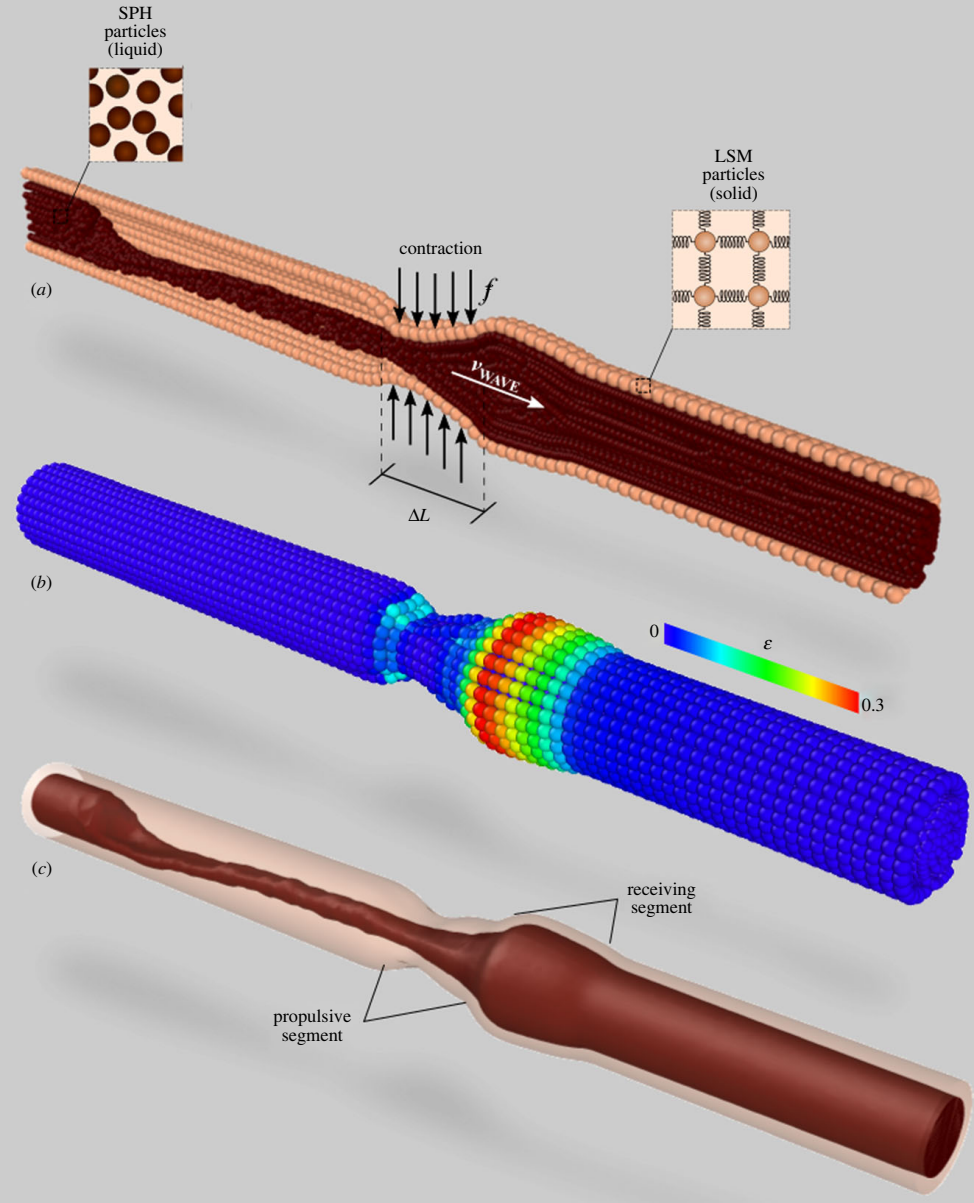
Particle representation of the discrete multiphysics model (*a*), Cauchy strain in the membrane (*b*) and continuum representation of the discrete model (*c*).

To simulate a peristaltic contraction, radial forces *f* are added to a section Δ*L* of the membrane (figure 1*a*). The position of Δ*L* moves along the tube with velocity *v*_WAVE_ replicating the peristaltic wave. As the contraction moves along the intestine, the chyme is pushed forward (from left to right in figure 1) and the membrane stretches to accommodate the advancing fluid. We call *v*_COM_ the displacement per unit time of the fluid's centre of mass, and *ɛ* the average stretch (strain) of the tube diameter (figure 1*b*). During the simulation, we measure *ɛ* since this is a controlling mechanism of peristalsis [19] that is going to play an important role in the next sections. During each contraction, the intestine can be divided into three regions (figure 1*c*). The first is the *propulsive segment*, where the intestine's circular muscles contracts; the second is the *receiving segment*, where muscles relax allowing the lumen to expand; the third is the rest of the tube that it is neither contracted nor relaxed. While Δ*L* moves along the tube, these three regions move accordingly.

**Figure 2.**
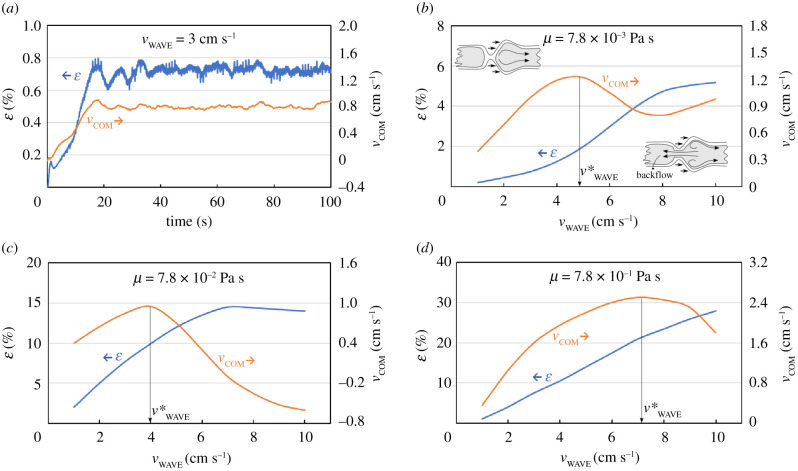
Propagation velocity of the luminal content (*v*_COM_) and stretch of the membrane (*ɛ*) versus time for *v*_WAVE_ = 3 cm s^−1^ and *μ* = 7.8 × 10^−3^ Pa s (*a*). Final values of *v*_COM_ and *ɛ* versus *v*_WAVE_ for *μ* = 7.8 × 10^−3^ Pa s (*b*), *μ* = 7.8 × 10^−2^ Pa s (*c*), and *μ* = 7.8 × 10^−1^ Pa s (*d*). The optimal wave velocity v*_WAVE_ maximizes *v*_COM_.

For the moment, we consider ‘static’ peristaltic waves where Δ*L* moves with constant velocity. Figure 2*a* shows a typical situation. After a quick transient, the luminal content moves at approximately constant velocity (*v*_COM_). At the same time, the stretch (*ɛ*) of the receiving segment also reaches a plateau. The final *v*_COM_ and *ɛ* depend on both the velocity of the peristaltic wave (*v*_WAVE_) and the viscosity of the luminal content (*μ*). The intestinal content can have a complex rheology [20], but, for simplicity, the fluid is considered Newtonian. We chose three viscosities (*μ* = 7.8 × 10^−3^, 7.8 × 10^−2^ and 7.8 × 10^−1^ Pa s) to cover a range consistent with the available data [21]. For each viscosity, we ran 10 simulations with *v*_WAVE_ between 1 and 10 cm s^−1^. Figure 2*b,c* shows the plateau values of *v*_COM_ and *ɛ* versus *v*_WAVE_ for different viscosities. In general, higher values of *v*_WAVE_ tend to increase *v*_COM_. However, if the wave moves too quickly, the propulsive segment has no time to close completely and some of the fluid leaks backwards resulting in lower *v*_COM_. Electronic supplementary material, video S1 shows the case of high viscosity and low velocity (*μ* = 7.8 × 10^−1^ Pa s, *v*_WAVE_ = 1 cm s^−1^), where the propulsive segment closes completely, and the fluid moves with the wave. Electronic supplementary material, video S2 shows the case of high viscosity and high velocity (*μ* = 7.8 × 10^−1^ Pa s, *v*_WAVE_ = 10 cm s^−1^), where the propulsive segment has no time to close completely and backflow is observed. The interplay between *v*_WAVE_ and *μ* produces various situations, which include the up-and-down profile of figure 2*b*, or even negative *v*_COM_ when the backflow is higher than the forward flow (figure 2*c*). Given the viscosity of the luminal content, there is an optimal v*_WAVE_ that maximizes transport (i.e. *v*_COM_); higher velocities are counterproductive because of backflow.

Current multiphysics models (e.g. [22–24]) only account for fixed *v*_WAVE_, whose propagation pattern is hardcoded and does not adapt to the luminal environment. However, this is not how our body works. The ENS adapts the peristaltic wave to the physical properties of the luminal content that changes continuously along with the gastrointestinal (GI) tract. ‘Intelligent’ models were developed coupling multiphysics with ANNs [11]. Nevertheless, these models do not provide a realistic representation of the ENS. In [10,11], for instance, the model could learn peristalsis, but it could not realistically adapt the contraction speed to the actual properties of the luminal content. This depends on how the ANN is coupled with the multiphysics model. In these studies, the ANN is ‘omniscient’. It has a perfect knowledge of the environment, which does not reflect how the ENS senses the luminal environment. In the next section, we develop an adaptive model that couples multiphysics with an ANN replicating the input–output relationship of the ENS.

### 2.2. Coupling the multiphysics model with the artificial neural network (adaptive model)

**Figure 3.**
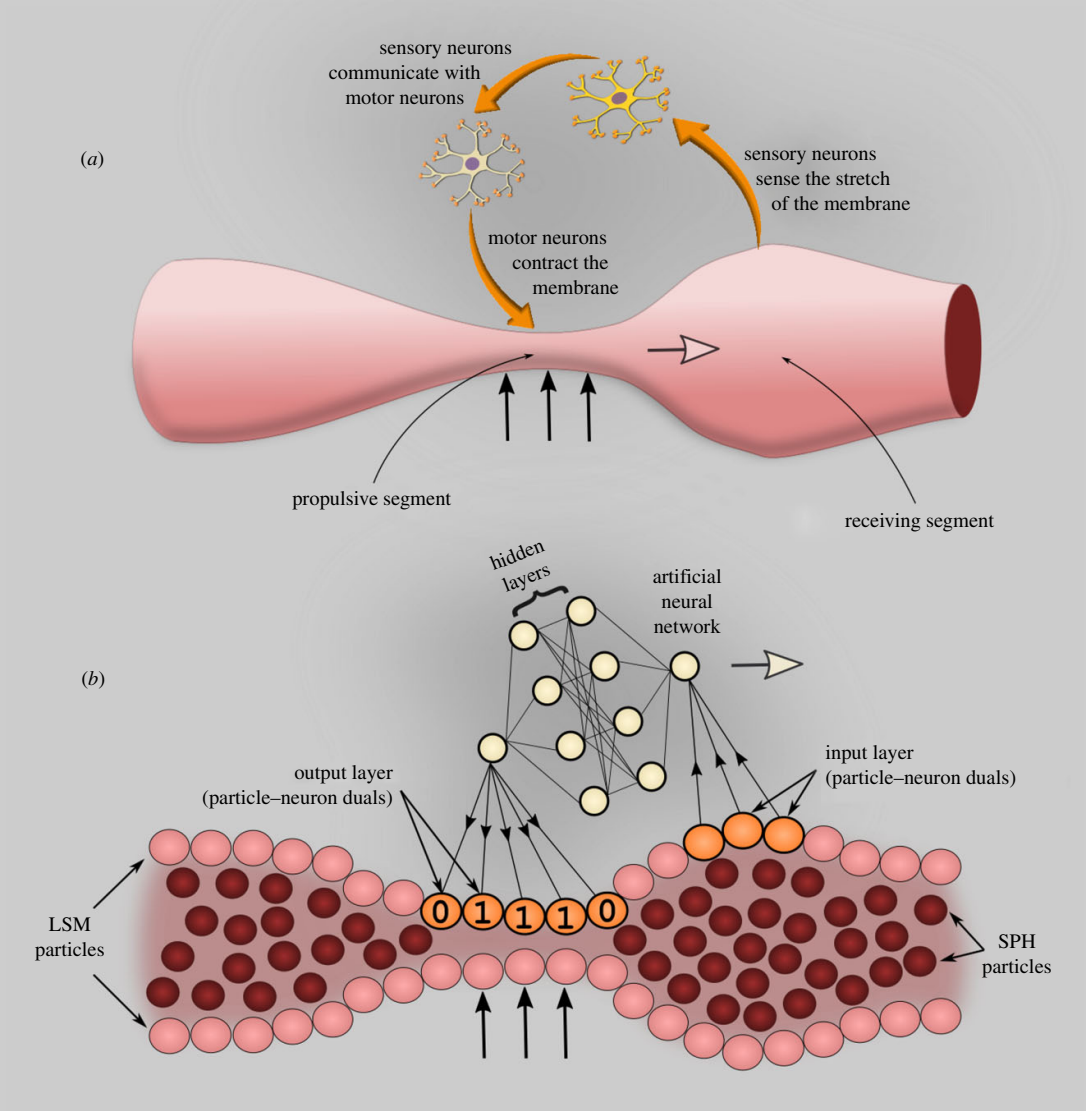
Interaction between sensory and motor neurons in the ENS according to the neuromechanical loop hypothesis (*a*), ANN replicating the input–output relationship of the ENS (*b*). The number of neurons/layers in the figure is only for illustrative purposes and does not represent the actual ANN. Details on the ANN architecture are given in the ‘Methods’ section.

The ENS contains around half a billion neurons embedded in the walls of the GI tract. It comprises several types of sensory and motor neurons that, besides peristalsis, coordinate blood flow, mucosal secretions and endocrine activity [25]. In this study, we only consider peristalsis and, in particular, high-amplitude propagating contractions (HAPCs), which transfer luminal contents over long distances. How the ENS controls HAPCs is still debated. Here, we employ the ‘neuromechanical loop’ hypothesis [26]. According to this hypothesis, the presence of the bolus stretches the membrane activating sensory neurons located on the receiving segment (figure 3*a*). These neurons communicate with motor neurons in the propulsive segment that contract the muscle layer around this segment. The ANN imitates this type of input–output structure (figure 3*b*). On the one hand, the LSM particles of the propulsive and receiving segment (orange particles in figure 3*b*) are computational particles used to model the elastic membrane. On the other hand, they are, respectively, the input and output layers of the ANN. These particles are *particle–neuron duals* [11], which allow for seamless communication between the DMP model and the ANN. They measure the stretch of the receiving segment and input this information into the ANN. The input layer converges to a pooling layer that calculates the average stretch. This information passes through the hidden layers. The final output of the ANN is the speed of the peristaltic wave (*v*_WAVE_). Based on *v*_WAVE_, the model calculates which part on the membrane should be contracted at any given time. A ‘1’ in figure 2*b* means that the section contracts (*f* applied to the particle). A ‘0’ means that the section does not contract. The ENS has several other functions in the body and its structure is more complex than this, but, in this study, we only focus on the stress/contraction relationship.

To save computational resources, instead of considering a large ANN spanning the entire tube, the ANN moves with the wave. The ANN slides over the membrane particles with velocity *v*_WAVE_ in such a way that input always corresponds to the receiving segment and output always corresponds to the contracting segment. The model measures the stretch *ε* of the receiving segment every 0.1 s. It feeds this information to the ANN that outputs the optimal *v*_WAVE_ maximizing mass transport (i.e. *v*_COM_). Initially, the ANN is not trained and, therefore, not capable of calculating the optimal *v*_WAVE_. This is not the typical classification problem that can be handled with supervised training but requires a different training approach called reinforcement learning (RL) [27]. Details on the ANN architecture, hyperparameters and training are given in the ‘Methods’ section.

### 2.3. The ‘healthy’ adaptive model

**Figure 4.**
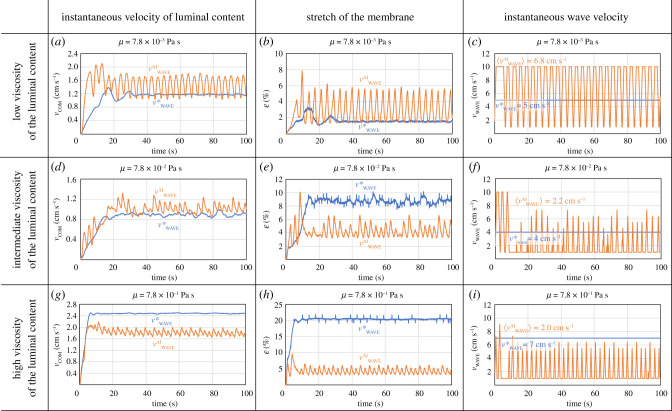
Comparison between *v*_COM_, *ɛ*, and *v*_WAVE_ calculated with the multiphysics model (blue lines) and the multiphysics + ANN model (orange lines). v*_WAVE_ is the optimal constant *v*_WAVE_ from figure 2.

Figure 4 compares the non-adaptive model (simple multiphysics) with the adaptive model (multiphysics + ANN) for *μ* = 7.8 × 10^−3^ (low viscosity), 7.8 × 10^−2^ (intermediate viscosity) and 7.8 × 10^−1^ Pa s (high viscosity). There are two levels of optimization here. The first is the optimal constant velocity wave from the simple multiphysics model (v*_WAVE_ in figure 2). The simple multiphysics model does not calculate v*_WAVE_ because it is non-adaptive. In figure 2, in fact, v*_WAVE_ was calculated ‘by hand’ from the data. The second is the optimization of a wave with non-constant *v*_WAVE_. The multiphysics + ANN model is adaptive and, therefore, not constrained to constant *v*_WAVE_. Each 0.1 s, it reassesses the situation and updates *v*_WAVE_. This is particularly important at low and intermediate viscosities. As an example, we can look at *μ* = 7.0 × 10^−3^ Pa s (low viscosity, figure 4*a*–*c*). The optimal speed of the constant velocity wave at low viscosity is v*_WAVE_ = 5 cm s^−1^ (figure 2*b*). With this *v*_WAVE_, the fluid moves with *v*_COM_ = 1.2 cm s^−1^ (figure 4*a*). If *v*_WAVE_ > v*_WAVE_, *v*_COM_ < 1.2 cm s^−1^ because of backflow. Instead of maintaining *v*_WAVE_ constant, the multiphysics + ANN model oscillates *v*_WAVE_ between 1 cm s^−1^ and 10 cm s^−1^ (figure 4*c*). It accelerates the fluid until the stretch of the receiving segment reaches 6% (figure 4*b*) and then reduces *v*_WAVE_ before backflow occurs. In this way, backflow is minimized, and the fluid is accelerated above *v*_COM_ = 1.2 cm s^−1^ (figure 4*a*).

At high viscosities, *v*_COM_ from the adaptive model is lower than that of the optimal constant velocity wave (figure 4*g*). At first glance, this looks odd. The explanation lies in the input–output structure of the ANN that mimics the ENS. An omniscient ANN would always find the optimal v*_WAVE_, but our ANN bases its decisions only on the stretch *ɛ* of the receiving segment. If we use the stretch as input, the ANN cannot always find v*_WAVE_. An easy way to see this is by comparing figure 2*c,d*. While in figure 2*c* high values of *v*_COM_ occur for *ɛ* < 12%, in figure 2*d* high values of *v*_COM_ are found for *ɛ* > 25%. These two conditions are incompatible. Therefore, the ANN, that only sees *ɛ*, cannot find a *v*_WAVE_ optimal for both cases, and settles for a trade-off between the two goals.

The solution adopted by AI keeps the stretch of the membrane low (*ɛ* < 6%) for most of the time (figure 4). This is also true for the actual ENS, which, above a certain stress, interrupts intestinal motility [28]. In the model, this outcome is possible only because we purposely designed the ANN with the same input–output structure as the ENS. This devise ensures that training of the ANN is constrained to solutions that are compatible with the way (based on measuring *ɛ*) the ENS perceives the position of the luminal content. Previous studies, where the ANN was designed differently (omniscient), did not achieve this result [11]. The input of the omniscient ANN was the actual position of the luminal content; the ANN converged to v*_WAVE_, which is optimal from an absolute point of view, but it does not keep *ɛ* low. Electronic supplementary material, videos S3 and S4 show AI in action. At low viscosities, it starts with a big push, which accelerated the fluid without excessively increasing the stress on the membrane (because of the lower fluid viscosity) and then settles for a lower velocity that reduces leakage (electronic supplementary material, video S3). At high viscosities, stress on the membrane is higher. Therefore, both the initial push and the final velocity tend to be slower (electronic supplementary material, video S4).

To validate the model, we compare 〈*v*^AI^_WAVE_〉 (the average *v*_WAVE_ calculated by the AI) with *in vivo* measurements. The HAPCs speed reported in the literature is given, depending on the variability of physiological data and different measurement techniques, in the range 0.2–12 cm s^−1^ [29] or at around 2 cm s^−1^ [30]. Furthermore, MRI analysis of the human caecum–ascending colon showed wave velocities of 0.98 and 2.2 cm s^−1^ at baseline and stimulated conditions, respectively [31]. Our simulations at intermediate (figure 4*f*) and high viscosities (figure 4*i*) are very close to [30]. The low viscosity case (figure 4*c*) shows a higher 〈*v*^AI^_WAVE_〉, which is within the range specified in [29]. Experiments with isolated rabbit distal colons [26] show that the peristaltic speed decreases with an increase in the viscosity. Numerical values are different in rabbits and humans, but the trend agrees with our model. As for the wave velocity, the 6% stretch threshold is also not hardcoded in the model, but the results of the training phase. This value is consistent with *in vivo* studies too. The threshold stretch to initiate peristalsis was measured in segments of the isolated guinea pig intestine [32]. For segments longer than 20 cm, the threshold is around 7%, which is close to the value determined by the model.

### 2.4. Modelling inflammatory bowel disease by adding digital inflammation to the adaptive model

In the previous section, we showed that, by making sure that the ANN mimics the structure of the ENS, the behaviour of the adaptive model is consistent with the behaviour of the real intestine. We trained the network to optimize transport, but the ANN also keeps the stress in the membrane low, which is exactly what occurs in the real intestine [28]. Therefore, it can be interesting assessing the reliability of the model in a variety of other scenarios. We add (digital) inflammation to the intestinal walls with the aim of inducing IBD to the model. IBD is an umbrella term for a group of inflammatory conditions of the intestine; it can have several causes, but one of the common consequences is inflammation of the intestinal walls. This inflammation triggers the sensory neurons on the membrane in such a way that the ENS perceives the membrane as more stretched than it actually is [33]. In the model, this is produced by ‘hacking’ the input layer of the ANN. The stretch of the membrane is a physical quantity; it does not change with inflammation. What changes is the perception of the neural network to the stretch. This false perception is achieved by adding a quantity of *ɛ_φ_* to *ɛ*. The degree of inflammation is defined as

2.1 $$\varphi =100\times \frac{\varepsilon_{\varphi }}{\varepsilon +\varepsilon_{\varphi }}.$$

**Figure 5.**
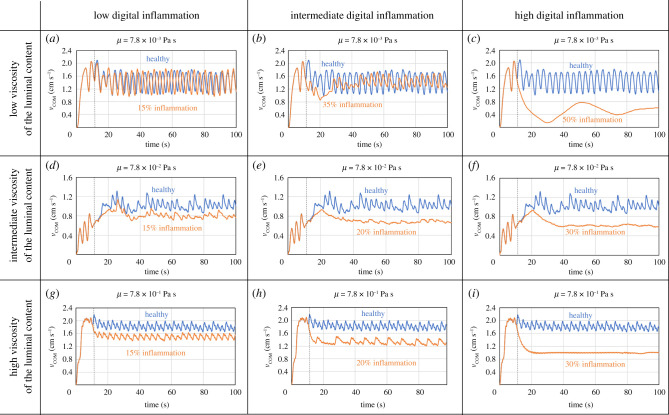
Comparison between healthy (blue lines) and inflamed (orange lines) models for different viscosities of the luminal content.

This, of course, is a very simplified approach to a complex phenomenon like inflammation. The model does not account for the aetiology of inflammation, but only for the effect that it has on the input layer of the ANN. Figure 5 shows how the system reacts to different degrees of inflammation in comparison with the healthy model. For all simulations, the initial 10 s correspond to a healthy section. After this, the wave moves into an inflamed section. Electronic supplementary material, video S5 shows an example (low viscosity): the first half of the tube is healthy, the second half has 50% inflammation. The presence of inflammation tends to slow down the wave bringing to longer transit times.

At first glance, this result looks counterintuitive. A typical consequence of IBD is diarrhoea, which implies shorter rather than longer transit times. However, recent studies found that subjects with IBD-related pathologies exhibit colon stasis (i.e. higher transit times) and diarrhoea is due to exudation of mucus and blood in the sigmoid and rectum rather than rapid transit times [33]. Therefore, the model captures this apparently counterintuitive behaviour correctly. It also captures the role of viscosity correctly. In fact, high-viscosity foods that produce higher stresses on the membranes are known to exacerbate IDB symptoms [34].

## 3. Methods

### 3.1. The discrete multiphysics model

The DMP model accounts for a flexible tube, representing the walls of the intestine, and a viscous fluid, representing the chyme (figure 1). In DMP, the domain is divided into computational particles that interact with each other by means of forces. If the forces acting on a particle mimic the elastic forces occurring in solids, the particle behaves like a solid; if they mimic the viscous and pressure forces occurring in liquids, the particle behaves like a liquid: the LSM is used for calculating elastic forces, SPH for viscous and pressure forces. Table 1 lists all the details of the model used in this study. The diameter of the tube is closer to the size of the colon (approx. 5 cm) than the small intestine (approx. 3 cm). Besides size, there are, of course, regional differences in how the GI tract senses and reacts to the luminal environment, but, in principle, the proposed computational approach can be used for both systems. The reader can refer to [17] for details on the SPH theory, and to [18] for the LSM theory; more details on the specific intestine DMP model can be found in [9].

**Table 1.** Technical details of the DMP model; the reader can refer to [9,17,18] for explanation of the items in the table that are not explicitly discussed in the text.

number of particles, *N*	14 578	solid 2500; liquid 12 078
mass of particles, *m*	2.5 × 10^−4^ kg	
elastic forces (LSM)	$F_{ij}=k(r_{ij}-r_{0})$	Hookean spring: *r_ij_* is the distance between particles *i* and *j*, *k* = 5 × 10^−1^ N m^−1^, *r*_0_ = 6 × 10^−3^ m
pressure (SPH)	$P=\frac{c_{0}\rho_{0}}{7}\left[{\left(\frac{\rho_{i}}{\rho_{0}}\right)}^{7}-1\right]$	Tait equation: *ρ_i_* is the density of particle *i*, *ρ*_0_ = 10^3^ kg m^−3^, *c*_0_ = 0.005–0.5 m s^−1^ (depending on viscosity)
viscous forces (SPH)	$\Pi_{ij}=-\alpha h\frac{c_{0}}{\rho_{ij}}\frac{v_{ij}r_{ij}}{r_{ij}^{2}+bh^{2}}$	*v_ij_* is the relative velocity between particle *i* and *j*, *ρ_ij_* = *ρ_j_* + *ρ_i_*, *h* = 1.2 × 10^−2^ m, *ρ* = 1, *b* = 0.01.
fluid–solid interaction	$U_{ij}=A\left[1+\cos\left(\frac{\pi r_{ij}}{r_{0}}\right)\right],\;r<r_{0}$	potential to avoid compenetration between solid and liquid particles: *A* = 2 × 10^−6^ J
boundary conditions	periodic	particles that exit from one side of the tube, re-enter from the other side
time step, Δ*t*	Δ*t* = 2 × 10^−3^ s	
length of the tube, *L*	0.63 m	
radius of the tube, *R*	0.025 m	
length of the contraction section, Δ*L*	0.063 m	the length of Δ*L* is fixed, but the position can be moved everywhere along the tube *L*
contraction force	*f* = 10^−3^ N	this force is added to each particle in the section Δ*L*

### 3.2. The artificial neural network and its training

There are 250 LSM particles in the section Δ*L*. These represent the input layer of the ANN. The input layer goes into a pooling layer that calculates the average stretch *ɛ* over the whole section. This information passes into two hidden layers (100 neurons each) and finally to the output layer. The output layer has three neurons, which correspond to three possible actions: (i) increase peristaltic speed by Δ*v* = 0.1 cm s^−1^, (ii) maintain the same velocity or (iii) decrease peristaltic speed by Δ*v*. Based on the new velocity, the section Δ*L* moves along the tube by a certain distance. Knowing the new position of Δ*L*, we can determine to which particles we need to exert the contracting force *f* for advancing the peristaltic wave. As mention in the ‘Results and Discussion’ section, the ANN requires training before working properly. This is not a classification problem and training is carried out with a technique known as RL [27]. Given the input (called *state* in RL), the ANN is trained to choose a specific *action* that maximizes a *reward*. For the problem under investigation, the input is *ɛ*, the possible actions are (i) increase, (ii) maintain or (iii) decrease *v*_WAVE_ by Δ*v*, and the reward is *v*_COM_. There are several RL algorithms available. Here, we use a method called cross-entropy [27]. The training phase is divided into *N* episodes constituted of *M* simulations (batches) each. During each episode, a random viscosity between *μ* = 7.8 × 10^−3^ Pa s and *μ* = 7.8 × 10^−1^ Pa s is chosen, and *M* simulations are carried out with this value of the viscosity. All simulations start at rest (*v*_WAVE_ = 0) and are carried out for 5 s. Every 0.1 s, the model measures *ɛ* and the ANN produces the probabilities of increasing, maintaining or reducing *v*_WAVE_. The new action is chosen randomly according to these probabilities. After the model executes the new action, the membrane will react with a new stretch, which will trigger a new action from the ANN, and so on. For each episode, we calculate the reward. Due to randomness in choosing the actions, some episodes will produce better rewards than others. The core of the cross-entropy method is to throw away bad episodes and train the ANN on better ones. We use the 70th percentile of all rewards, which means we only keep the 30% of the simulations with the highest reward and throw away all other episodes. We conclude the episode by training the ANN with these ‘elite’ episodes. The new episode is carried out in the same way and will produce new training data for the network. With each episode, the ANN learns how to repeat the best actions, which leads to higher and higher rewards. Details on the ANN and the training are given in table 2. The reader can refer to the Keras documentation [35] for the items in table 2 not explicitly discussed in the text. Training does not always converge to an optimal solution (called *policy* in RL), and even when it does, there is no a clear criterion for deciding if it is an absolute or a relative minimum of the loss function. In this study, we repeated training four times and compare the final models against experiments and *in vivo* measurements. As discussed in the ‘Results and Discussion’ section, the fourth model was chosen as the more realistic since (i) peristaltic velocity matches the real value, (ii) the trend viscosity-versus-*v*_WAVE_ is correct, (iii) it properly avoids high *ɛ* and (iv) remains reliable when digital inflammation is added.

**Table 2.** Architecture of the ANN and hyperparameters used for training; the reader can refer to [35] for the items in table not explicitly discussed in the text.

input layer	*N* = 250	
pooling layer	input 250, output = 1	performs average
hidden layer 1	*N* = 100	*φ* = relu
hidden layer 2	*N* = 100	*φ* = relu
output layer	*N* = 3	*φ* = softmax
hyperparameters	loss = categorical cross-entropy	
	metrics = accuracy	optimizer = adam
	batch size = 10	episodes = 20 000

### 3.3. Software

The simulations of the physical model are carried out with the open-source software LAMMPS [36] compiled as a Python library, while the Python library Keras [35] is used for training the network. Visualization and videos were processed with the software Ovito [37].

## 4. Conclusion

This study proposes a technique for coupling multiphysics and ANN to design *in silico* models of human organs that autonomously adapt their behaviour to environmental stimuli. The technique is applied to a model of the intestine that, based on the stretch of the membrane, regulates its peristaltic contractions to optimize transport. multiphysics ensures that the model complies with the mechanics of the system, while the neural network is trained to replicate the action of the ENS within the constraints imposed by the mechanics. A traditional approach would require hardcoding in the model the functional relationship between the stretch threshold that initiates contraction and the velocity of the peristatic wave. Here, they spontaneously emerge as a result of the training process. After training, we only need to validate the model by verifying that these parameters are consistent with *in vivo* measurements. The model has another unexpected property. It responds to atypical variations of its functioning with ‘symptoms’ that reflect those arising in real diseases. We started with a healthy intestine model and we made it artificially ill by adding digital inflammation. As a result, the model motility patterns are disrupted in a way that mirrors inflammatory pathologies like IBD. This seminal paper targets peristalsis, which represents only a subset of intestinal motility, and only a specific aspect of IBD. However, it brings us one step closer to a virtual physiological human that not only reproduces the mechanics of our body, but also adjusts its behaviour to environmental factors. This includes the possibility of computer models that, under certain conditions, can ‘become ill’ with symptoms that simulate those of real diseases.

## Data accessibility

All LAMMPS input files and Python code used in the simulations are freely available under the GNU. General Public License v3 and can be downloaded from the repository edata.bham.ac.uk/570/.
